# First Case of Enterovesical Fistula Caused by Ischaemic Enteritis

**DOI:** 10.7759/cureus.16452

**Published:** 2021-07-18

**Authors:** Uma Pradhan, Ravi Kumar, Prem Narayan Agarwal, Gulshanjit Singh, Piyush Puri

**Affiliations:** 1 Surgery, Shree Guru Gobind Singh Tricentenary (SGT) Medical College, Gurgaon, IND; 2 General Surgery, Shree Guru Gobind Singh Tricentenary (SGT) Medical College, Gurugram, IND; 3 Critical Care Medicine, Rama Medical College Hospital & Research Center, Hapur, IND

**Keywords:** enterovesical fistula, fistula, pneumaturia, tuberculosis, ischaemic enteritis, ibd, crohns, ischemic colitis

## Abstract

We present the case of an enterovesical fistula (EVF) caused by ischemic enteritis. Ischemic enteritis is caused either by mesenteric macrovasculature occlusion or any pathophysiologic vasospasm of the microvasculature. In other words, ischemic enteritis (IE) occurs when the inflow of blood to the small intestines is reduced. The frequency of ischemic enteritis is less as compared to ischemic colitis because of the vast blood supply to the small intestine. It is the first case to be reported in the medical literature to date. EVF is a rare entity. It is a pathological connection between the bowel loops and the urinary bladder. EVF is a result of an underlying disease or injury. EVF is mostly caused by diverticular diseases, carcinoma colon, Crohn's, and inflammatory bowel disease, iatrogenic, appendicitis, carcinoma cervix, etc. Due to the formation of this abnormal connection, contents of the intestines, including the air, food content, etc., are usually found in the urine. Patients usually present with the complaint of irritative urinary tract symptoms and recurrent urinary tract infection (UTI). Surgical management is the mainstay of treatment although medical management is tried for those who cannot bear to undergo surgery.

## Introduction

Enterovesical fistula (EVF) represents an abnormal communication between the intestine and bladder. Causes of EVF are plenty, but they have been devised into five main classes: congenital, traumatic, tumor, inflammatory, and others. In the Western world, the main cause is diverticulitis (56.3%) and almost all the cases are associated with colonic or bladder fistula. The second most frequent cause in the Western world is malignant tumors, which are located mainly in the intestine (20.1 %). Other associated tumors include bladder, cervical, ovarian, and prostate cancers and non-Hodgkin's lymphoma of the small intestine. The third most prevalent cause is Crohn’s disease (9.1%), which occurs mainly in the ileum. Other causes include iatrogenic injury (3.2%); trauma; foreign bodies in the intestinal tract; radiotherapy; chronic appendicitis; tuberculosis; and syphilis. The male-to-female ratio is 3: 1. The lower prevalence in females is owing to the interposition of the uterus between the bladder and sigmoid colon, but higher rates of both colovesical and colovaginal fistula have been reported in females who have previously undergone a hysterectomy [1]. Diagnosis can be challenging and thus can be delayed [2]. Treatment is based on the etiology of EVF [3]. It is not a very common entity encountered by surgeons in clinical practice and to the best of our knowledge, there is no published case of enterovesical fistula (EVF) due to ischemic enteritis.

## Case presentation

A 34-year-old male, carpenter by occupation, presented to the hospital with complaints of yellowish discoloration and foul-smelling urine with debris, pneumaturia, and burning micturition for the last 10 days. He had a history of recurrent urinary tract infections (UTIs) for which he was treated previously at the rural health care center. He also had multiple episodes of pain in the right lower abdomen for which he used to take over-the-counter (OTC) drugs and had on-off episodes of constipation. Subsequently, he was diagnosed with abdominal tuberculosis, and it was treated with antitubercular drugs for a period of six months. He had no history of diverticulitis, inflammatory bowel disease (IBD), and any surgery. The only significant medical history was Koch's abdomen and a history of smoking for the last 10-12 years, which is a risk factor for ischemic enteritis. Cartridge-based nucleic acid amplification test (CB-NAAT) was done, which was negative for tuberculosis. Lab investigations were within the normal limit except for urine microscopy, which showed numerous WBCs and epithelial and pus cells. Urine culture had led to the growth of mixed organisms including Escherichia (E.) coli.

Due to recurrent episodes of UTI, ultrasonography (USG) was advised, considering his economically weak background. USG was done, which suggested a heterogeneous wall lesion of the urinary bladder in the dome suspicious of a urachal mass. No calculi were seen on the USG.

**Figure 1:**
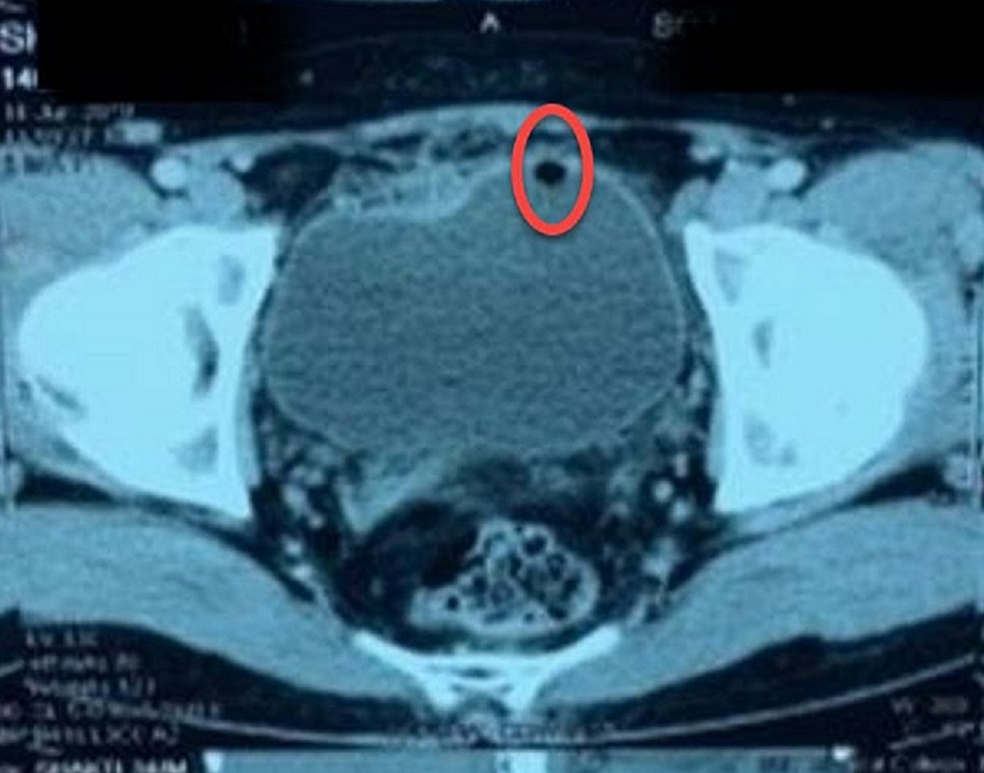
CT scan showing an air bubble in the bladder

Considering the USG findings, a further CT scan was suggested, which showed an air bubble in the urinary bladder, and he was diagnosed with EVF (Figure 1).

**Figure 2:**
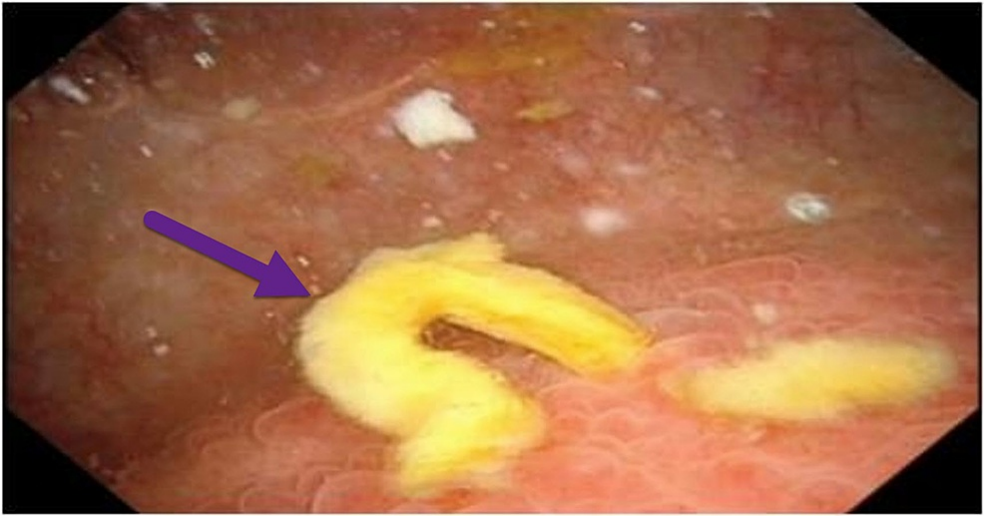
Cystoscopy showing fecal matter, which is found in the urinary bladder due to the abnormal connection

To look out for any neoplastic masses in the urinary bladder, diagnostic cystoscopy was done. Cystoscopy was suggestive of fecal matter with an irregular area around 2 cm at the fundus, near the midline. Since no masses were seen, neoplasia was ruled out (Figure 2).

Due to the history of Koch's abdomen, it has been one of the major differentials for intravesical fistula. And since it is fairly widespread in India, it has been thought to be the cause of EVF.

**Figure 3:**
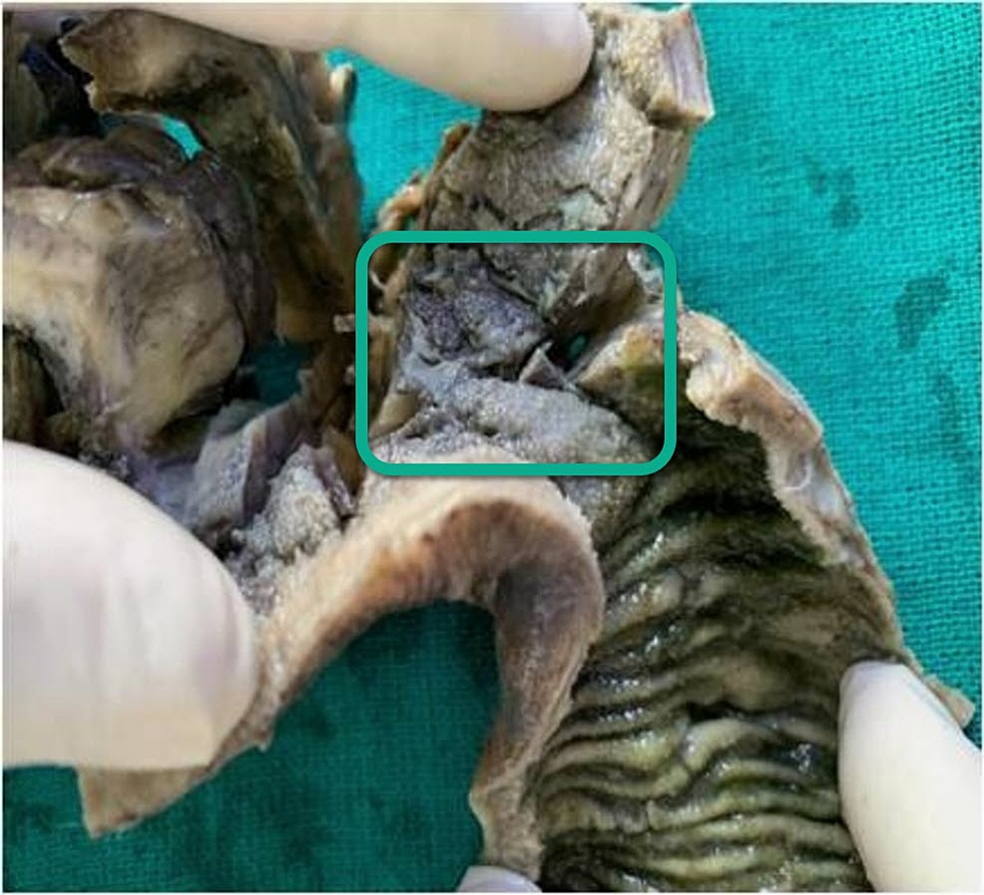
Resected bowel with fistulous communication to the bladder

**Figure 4:**
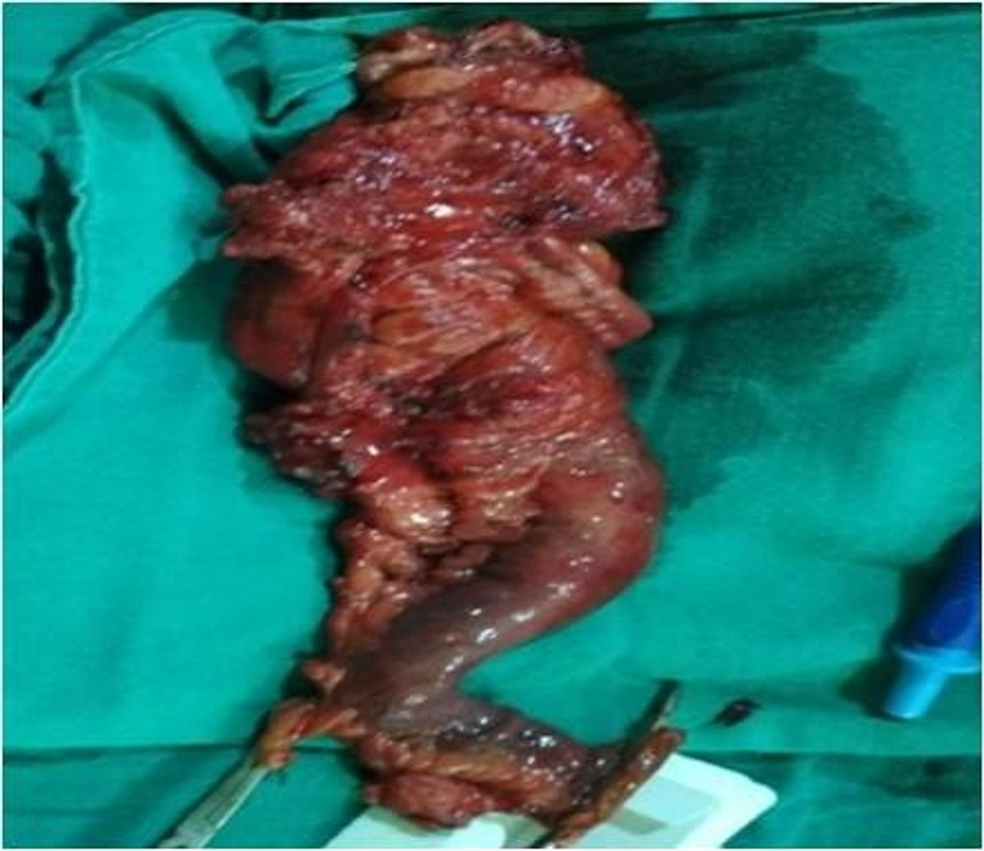
Resected ileocecal junction

The radiologist confirmed the fistulous tract arising from the ileum. Considering the clinical symptoms and using radiological imaging, a diagnosis of EVF was made. Ileocolonoscopy was not performed because it was expensive for the patient to bear the cost of extra investigations and minimally informative since CB-NAAT for tuberculosis was already negative, and there was no history related to any bowel disorder. Hence, explorative laparotomy was planned and the findings were: Inflamed and thickened ileocecal junction, communication between urinary bladder and ileum, and enlarged lymph nodes. Limited resection of the ileocecal junction with ileo-ascending anastomosis and repair of the urinary bladder was done (Figures 3-4). The postoperative period was uneventful except for a minor surgical site infection, which was taken care of. He was Foley's catheterized for 14 days at the hospital and discharged on the fifteenth day with complete recovery.

**Figure 5:**
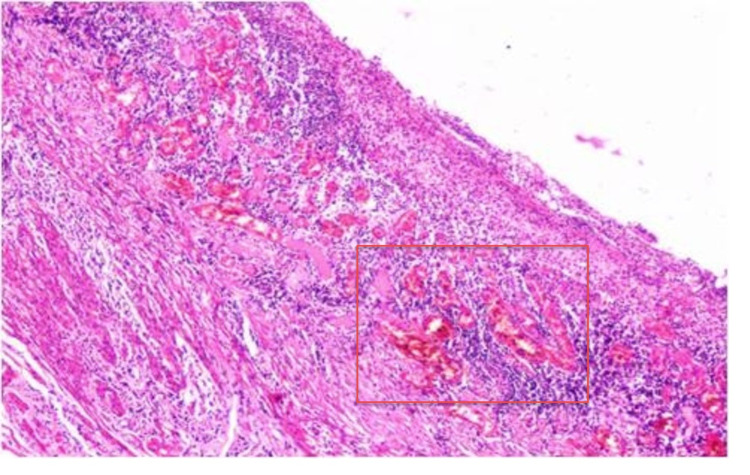
Histopathology showing the necrosed area

In addition, the tissue of the exploratory laparotomy was sent for histopathologic examination. Since tuberculosis is highly prevalent in India, we expected it to be the cause of the fistula, with a granuloma with Langhans-type giant cells. Instead, the histopathology report showed ileocecal stricture. Section of the ileocecal segment reveals acute enteritis and mucosal ischemic necrosis. The appendix showed serositis, and lymph nodes showed extensive areas of hemorrhage. Histology showed areas of congestion, hemorrhage, and necrosis extending up to the mucosal layer. Loss of villi is also noted along with inflammatory cells, suggesting ischemic etiology (Figure 5).

## Discussion

EVF is a rare entity that has various causes. The most common cause is diverticulitis followed by malignant tumors, especially of the colon. The third most prevalent cause is Crohn’s disease, which mainly involves the ileum. Other less common causes of EVF included radiation exposure, iatrogenic injuries, especially in pelvic surgeries, and foreign bodies are rare causes. 

Gouverneur’s syndrome characterized by suprapubic pain, frequency, dysuria, and tenesmus is the main hallmark of EVF [4]. Other clinical features like pneumaturia, debris in the urine, recurrent UTI, etc. can also be present.

On clinical suspicion of EVF, cystoscopy is used to initially diagnose fistulae in 30%-50% of cases. Prior to advances in radiology diagnostic techniques, cystoscopy was considered the most reliable method of diagnosis. On cystoscopy, a congested erythematous urothelium is observed, with a mucosal edematous lining in the center emitting mucus and gas. Cystoscopy is performed for identifying the defect and its position and for biopsy. Treatment is done according to etiology. Diagnostic CT accuracy for colovesical fistula detection can be up to 90-100% [2,5-6]. CT scans usually show intravesical air (90%), focal bladder wall thickening (90%), adjacent bowel wall thickening (85%), and passage of contrast medium into the bladder administered rectally or orally (20%) [7]. MRI can exactly delineate the fistulous tract and its features like its complexity, whether single or multiple tracts, without any need for intra-luminal contrast, but it could be expensive and more time-consuming. Any previous medical history of surgeries, bowel habits, etc. can also help in the diagnosis.

Nonoperative treatment is particularly useful in cases of Crohn's disease. Medical therapy usually includes bowel rest, total parenteral nutrition, steroids, antibiotics, and urethral catheter drainage. Nonsurgical management of the EVF is usually done for those patients who are unfit for extensive surgeries or have a malignant neoplasm with extensive spread. Currently, surgical treatment consists of single-stage resection of the diseased bowel with anastomosis, and primary closure of the bladder defect remains the mainstay of the treatment.

Various risk factors that could be hampering the blood flow and leading to ischemia in the gut are atherosclerosis, hypertension, diabetes mellitus, increasing age, alcohol consumption, arterial embolus, hypercoagulable states, severe dehydration, smoking, etc.; they are also some of the risk factors associated with ischemic enteritis [8].

In this case, the patient had an ileocecal fistula because of ischemic enteritis. This is a rare cause of this entity, which was treated with resection and ileo-ascending anastomosis.

## Conclusions

EVF, which is a rare entity, has even rarer causes for it. Due to the lack of randomized control trials and the rarity of the condition, no proper diagnostic and treatment guidelines exist. This ailment remains an area of interest for the doctors to devise proper protocols for the better prognosis and outcomes of this condition. The management of EVF is primarily dependent on the underlying pathology, the site of the intestinal lesion, and the patient's preoperative status. Single-stage surgery is the preferred option in most cases.
